# Cushion hypothesis and credit risk: Islamic versus conventional banks from the MENA region

**DOI:** 10.1371/journal.pone.0306901

**Published:** 2024-07-22

**Authors:** Islam Abdeljawad, Mamunur Rashid, Muiz Abu Alia, Rana Qushtom, Mahmoud Irshaid, Ahmad Sahyouni

**Affiliations:** 1 Finance and Banking Department, An-Najah National University, Nablus, Palestine; 2 Christ Church Business School, Canterbury Christ Church University, Canterbury, United Kingdom; 3 Accounting Department, An-Najah National University, Nablus, Palestine; 4 Department of Sharia and Islamic Banks, An-Najah National University, Nablus, Palestine; 5 Higher Institute for Administrative Development, Damascus University, Damascus, Syria; MY University Islamad, PAKISTAN

## Abstract

Conventional banks are ‘indirectly’ allowed to take more risk under the shadow of sovereign guarantees. Banks commit moral hazards as any major banking crisis will be ‘cushioned’ by deposit insurance and bailed out using the taxpayer’s money. This study offers an alternative explanation for the determinants of banks’ credit risk, particularly those from the Islamic regions. Although conventional banks and Islamic banks may share state and social cushioning systems, Islamic banks are strictly prohibited by moral and religious principles from gambling with depositors’ funds, even if there is a cushion available to bail them out. However, banks belonging to collective societies, such as those in the MENA area, may be inclined to take more risks due to the perception of having a larger safety net to protect them in the event of failure. We analyse these theoretical intersections by utilising a dataset consisting of 320 banks from 20 countries, covering the time span from 2006 to 2021. Our analysis employs a combination of Ordinary Least Squares (OLS), Fixed Effects (FE), and 2-step System-GMM methodologies. Our analysis reveals that Islamic banks are less exposed to credit risk compared to conventional banks. We contend that the stricter ethical and moral ground and multi-layer monitoring system amid protracted geopolitical and post-pandemic crises impacting Islamic countries contribute to the lower credit risk. We examine the consequences for credit and liquidity management in Islamic banks and the risk management strategies employed by Islamic banks, which can serve as a valuable reference for other banks.

## 1. Introduction

Credit earns the highest income for the banks. Agency theory explains that bank managers are obligated to disburse more credit to satisfy banks’ appetite to earn more returns for the owners and incentives for the managers. Therefore, larger banks with better profitability, liquidity, and capital reserves will find ways to disburse more credit [1–3]. Financially weaker banks must disburse credit to stay afloat, survive competition, and maintain going concern [4]. Despite higher credit risk, banks may still disburse higher credit due to several market imperfections, such as the tax-leverage nexus and overconfident management’s behaviour towards risk [5,6].

The above approaches to bank credit risk from the extant literature have been puzzling, especially with an increase in such risks despite several minor and major global crises, including the global financial crisis of 2007–08. Accepted in Basel II, credit risk is "*the potential that a bank borrower or counterparty will fail to meet its obligations following the agreed term*" [7]. By this definition, on the one hand, banks adversely select clients that are risky but offer a high return. On the other hand, banks commit moral hazards as any major banking crisis will be ‘cushioned’ by deposit insurance and bailed out using the taxpayer’s money. Hence, we offer two propositions. First, banks are ‘indirectly’ allowed to take more risk under the shadow of sovereign guarantees. Second, in line with Buckley et al. [8], a firm’s risk exposure depends on its ability to mitigate those risks. Therefore, larger, more profitable, and more liquid banks with a higher capital base find higher incentives for taking risks [9].

In line with these propositions, this study offers an alternative explanation of the factors affecting the credit risk of the banks, particularly those from the Middle East and North Africa (MENA) region. The Cushion Hypothesis, which is primarily found in research on personal financial risk-taking, explains the positive influence of the availability of safety nets that encourage people to take extra risks [10]. These safety nets can take different forms in an institutional setting. For example, alongside positive asset and profit growth [11], a firm may take more risk if the economic condition—say, in terms of the growth of gross domestic products—is good [12]. Banks might be less reluctant to proactively identify and manage risk in the presence of a strong derivative market [13]. Also, poor regulatory quality encourages excessive risk-taking [14].

Islamic banks have a multi-layer monitoring system that includes internal standard operating procedures, Shariah screening, an internal Shariah advisory board, internal and external auditors, central Shariah supervision, and monitoring by the Central bank. The core objective of these actors is to ensure that Islamic banks follow Islamic Shariah rules, do not invest in excessively risky instruments, and do not gamble [15,16]. Hence, irrespective of the cushioning system, Islamic banks will opt for less risky credit than conventional banks due to their restrictive Islamic principles, though Islamic asset-based or profit-loss-sharing-based financing contracts carry higher inherent cost [15].

This study examines determinants of credit risk among banks in the Middle East and North Africa (MENA) region. According to the IFSB [17], MENA countries, when coupled with the Gulf Cooperation Council (GCC) region, hold over 60% of the global $2.10 trillion Islamic finance market. MENA countries have also seen significant growth in private sector credit due to the privatization drive emphasized in the Vision statements of multiple oil-rich countries, such as the Saudi Vision of 2030, the Qatar Vision of 2030, and the Bahrain Vision of 2030. MENA countries offer unique legal, social, and economic systems compared to other top Islamic finance markets in Southeast or South Asian regions. Countries in the MENA region have been going through several severe political events that have reshaped their economies and social environments [18].

This study considered data from a panel of 320 banks across 20 countries for the period 2006–2021. The data was analysed using OLS, FE, and 2-step system-GMM approaches. Results indicate that on average, 10.1% of the loans (4% of the assets) were non-performing. Large banks with higher profitability and capital adequacy had lower credit risks. Banks from countries that experienced high GDP growth rates and higher regulatory quality reported lower credit risk. Islamic banks had lower credit risks than conventional banks, and their profitability, liquidity, and asset quality were able to restrict these risks (an efficient operational cushion) better than conventional banks.

The impact of these findings is significant for bankers and policymakers. We contribute to two strands of literature. The first group of literature defames credit risk as the most crucial risk for the banking sector that may spread to the bank’s capital, creating an insolvency problem [19,20]. The second group of literature identifies credit risk as a function of agency problems [21]; as such, the tendency to ‘build an empire’, to win the competition, or to maximize personal incentives tied to bank performance determines higher credit risk [5,22]. We argue that it is the direct and indirect safety nets that banks enjoy in different markets that drive credit risk. We provide a counterargument to Ahmed [23] and support the results of Zarrouk [24], as Islamic banks are less exposed to credit risk than conventional banks due to limited cushioning and restrictive internal operational procedures [25].

Policymakers should consider the persistence of the credit risk (significant lagged value) across time and that poor asset quality contributed significantly positively to the credit risk. The persistence of credit risk signifies that the conventional banks will continue to invest in risky credit until a major crisis or new regulatory scrutiny stops them. The moral hazard of "too big to fail" works as the best ‘cushion’ that is indirectly encouraging conventional banks to take more risks. Despite having a strong state-level cushion, Islamic banks were restricted in their risk-taking. Depositors seeking safer investment may choose Islamic banks due to lower exposure to risk. Relatively lower non-performing loan should also reduce overall cost of financing for Islamic loans, which is a positive signal for the borrowers of Islamic credits.

We discuss theory, literature, and hypotheses in Section 2. Section 3 reports methodology and data. Section 4 summarizes the findings. Section 5 concludes the study.

## 2. Literature review and the hypotheses development

### 2.1 Theoretical perspectives

According to Saeed et al. [15], the banking industry plays a crucial role in the advancement of the financial sector and the expansion of new investment opportunities for economic growth. Nevertheless, there are certain risks that pose a threat to the survival of banks in the foreseeable future. According to several sources [16], a significant proportion of these threats can be attributed to inadequate credit risk management and an inaccurate assessment of anticipated default risk. The concept of credit risk pertains to the evaluation of the likelihood of borrowers failing to fulfil their obligations to the banks [26,27]. This failure holds considerable sway over the stability of the banking system [28]. Therefore, regardless of whether they are Islamic or conventional, banks are susceptible to credit risks [29].

According to Safiullah and Shamsuddin [9], credit payments that are delayed for an average of over 90 days are commonly regarded as non-performing loans (NPL). An elevated proportion of NPL is indicative of increased credit risk. The management of credit risk holds significant importance for Islamic banks owing to the distinctive contractual association between credit customers and these banks. This is due to the application of a participatory financing approach by Islamic banks, which regards the credit client as a ’partner’ [30].

A comprehensive body of literature delves into the impact of a conservative religious and cultural outlook on financial risk-taking. The domain of Islamic finance and economics is characterized by a cautious management strategy that is founded on several levels of supervision [31]. Islamic countries’ socioeconomic and institutional frameworks are imbued with religiously restrictive norms. Islamic banks are anticipated to establish an institutional protective system that not only mitigates credit risk exposure but also minimizes associated agency costs. Islamic banks utilize a moral framework to enhance their standard operating procedures, such as credit matrix and risk management, with the aim of mitigating agency issues and signalling to the market their stability [4]. Islamic banks adhere to a consistent moral framework that prohibits the dissemination of inaccurate information and the provision of substandard credit by their bankers [32,33].

Islamic societies are typically characterized by a collective nature that places significant emphasis on the establishment of *Adl* (justice) and *Ihsaan* (excellence through righteousness) while simultaneously restricting unhealthy competition [31]. In light of this context, advocates of the Cushion hypothesis posit that collective societies exhibit elevated levels of cushions, potentially leading to the development of a perception of social and state cushioning whereby individuals believe that they will receive support if they fail [34–36]. Due to the presence of such protective measures, individuals tend to engage in riskier behaviours, as such, large vehicles are perceived as a means of protection in the event of a road accident [37]. These cushions serve as direct incentives for individuals to engage in risk-taking behaviour [38]. According to Weber and Milliman [36], cushions are typically characterized as protective measures that aid individuals in overcoming obstacles or preventing financial setbacks. The social cushion refers to the system of social support that is prevalent in collective societies, wherein a norm of collective assistance is activated in the event of a member of the society facing difficulties [35]. According to Schneider et al. [34], state cushion refers to the assistance provided by the government or state to aid individuals in recovering from unforeseen setbacks.

The cushioning system observed in Islamic societies may appear paradoxical. Islamic institutions are constrained from engaging in excessive and non-random risk-taking, as noted by several scholars [31,39,40]. Despite assuming conventional business risk while providing credit to specific enterprises, the determination undergoes multiple internal and external screening mechanisms. Hence, there exist ethical justifications and Islamic doctrines that oppose the customary societal and governmental support mechanisms accessible to traditional establishments. To clarify, it can be posited that Islamic financial institutions are likely to exhibit reduced credit risk due to the absence of any motivation to engage in the provision of high-risk loans. Most MENA societies tend to exhibit a collective nature, wherein both conventional and Islamic banks have access to state and policy-level safeguards, such as the deposit insurance program and Kafalah system for the Islamic bank credit [41]. Therefore, unless similar safety nets are monitored, Islamic banks may lose the benefit of moral and ethical advantages, which may lead to higher credit risk.

The stance towards credit risk management within Islamic banks is contingent upon the extent to which a prospective risk can be alleviated through operational efficacy. The credit derivative market in Islamic banks is currently not well established. In accordance with Shariah law, Islamic banks employ various measures such as Shariah screening, risk filtering, and pre-approval Shariah risk supervision to effectively manage risk. This has been highlighted in previous studies [1,42]. Therefore, Islamic beliefs and values serve as the sole mechanism for mitigating risk in the Islamic system, leading to a superior quality of credits characterized by a low level of non-performing loans (NPL).

The relationship between finance and growth has been found to be interdependent, with various factors such as the credit market, stock market, economic growth, bank competition, and entrepreneurship development being closely linked [22]. Following the global financial crisis of 2007–2008, there has been growing concern regarding the relationship between financial sector instability and institutional mismanagement of credits within the efficiency-finance-stability nexus. This includes the impact of factors such as (in)sufficient bank capital, (in)competent board members, and poor risk management and supervision during the global financial crisis, as highlighted by Berger and Bouwman [43], and Legowo and Gimpalas [44].

Agency theory posits that the governance system of banks exerts a significant influence on their risk-taking behaviour, with institutional factors being a key consideration. According to Jensen [21], managers have a proclivity to engage in "empire building" by utilizing surplus cash from free cash flow, which may lead to a more lenient credit policy. Bank managers who exhibit overconfidence tend to engage in excessive investment activities when they have substantial cash reserves at their disposal. In this context, it is possible for managers to grant large loans without taking into account the credit scores of their clients, with the objective of expanding their business operations. Consequently, as per Mwaurah’s [5] findings, the likelihood of defaulting on debts will rise proportionally, despite the bank’s size and profitability.

### 2.2 Bank-specific determinants of credit risk

The present study aims to investigate the relationship between bank credit and economic growth, bank efficiency, and financial stability. Although risk-taking attitude may have an influence on ultimate risk exposure, this study intends to focus on credit risk exposure rather than the risk management process. To achieve this objective, the study will identify a range of country-specific economic, bank-specific financial, and non-financial determinants of credit risk. These determinants will be discussed in detail in the subsequent sections. The credit risk of banks, being global institutions, is subject to the impact of both bank-specific and country-specific factors, as noted by Gulati et al. [45], Hashem and Abdeljawad [16], and Antony and Suresh [46].

#### 2.2.1 Bank size

Smaller banks are susceptible to differing levels of credit risk due to their predominant engagement with smaller clients. These banks possess a lower cash reserve, which limits their ability to adopt a lenient credit policy [47]. Thus, it can be inferred that smaller banks may face a higher level of credit risk. Antony and Suresh [46] found a negative relationship between bank size and credit risk. Several studies have observed that banks tend to assume greater credit risk as their asset size increases [11,48,49]. However, financial institutions of large size are associated with greater transactions and offerings that could result in elevated credit risk [50]. The extensive range of operations and products offered by large banks may result in a loss of control, thereby increasing the level of risk. Consistent with this view, Swami et al. [51], and Muhammed et al. [3] pointed out that bank’s size is positively linked with risks.

Larger banks have the potential to reap the rewards of economies of scale, which can enable them to diversify credit risk [52]. As a result, according to numerous studies [1,53,54], there is a negative correlation between a bank’s size and its credit risk. Islamic banks are generally smaller in size compared to the conventional banks. Therefore, credit risk in Islamic banks might be higher due to limited economies of scale. Organizations undertake greater risks when they are able to effectively manage them through the use of proactive and reactive measures. Large corporations are able to utilize risk management and hedging resources in order to minimize potential risks [8]. Thus, considering the ability of large financial institutions to alleviate and distribute credit risk, we posit that:

H_1_: Large banks, irrespective of their type, have lower credit risk. The negative effect is stronger for Islamic banks

#### 2.2.2 Profitability

Profitability is the return on investment that a bank generates across all of its investments. A higher level of bank profitability is indicative of effective credit management and suggests that the resulting earnings will be utilized for the purpose of improving credit performance through reinvestment. Although credit is a main source of bank’s income, it exposes the banks to credit risk as there is a possibility of non-payment by the borrowers [42]. In their study, Kabir et al. [2] utilized return on assets (ROA) as a measure of profitability and observed an inverse correlation between ROA and non-performing loans in both Islamic and conventional banking sectors. A negative relationship is supported by previous studies [46,51,54,55].

Islam [56] indicated that banks with profit efficiency are more successful in managing credit risk. Profitable Islamic banks may face increasing scrutiny from the Shariah board before new investment. Therefore, despite having higher profit, Islamic banks may experience lower credit risk. On the other side, it has been suggested that over a prolonged period of time, increased profitability could potentially build a cushion that can alter the risk tolerance of banks, resulting in elevated credit risk [6]. Although infrequent, prior research has also indicated that profitability has no significant influence on a bank’s credit risk [57]. In light of the risk provision policy, it is possible for banks that generate significant profits to maintain a substantial investment risk reserve, which can serve as a protective measure against potential risks. Thus, it is postulated that:

H_2_: Highly profitable banks–Islamic or conventional—have lower credit risks. The negative effect is stronger for Islamic banks.

#### 2.2.3. Liquidity

Bank liquidity refers to the reserve of funds that can be easily used to fulfil financial obligations as they arise [58]. Effectively managing liquidity risk ensures generating a high-quality, stable, large, and growing flow of net interest income for banks [19]. The minimum regulatory requirement for a bank’s liquidity is a crucial aspect to consider, as any discrepancy in the dollar amount and/or maturity between the assets and liabilities can potentially lead to a liquidity crisis. Banks that exhibit robust liquidity levels are indicative of a seamless inflow of deposits, which can be allocated towards credit investments. Consequently, there exists a relationship between bank liquidity and decreased credit risk, as posited by Safiullah and Shamsuddin [9]. Banks with a higher liquidity requirement may exercise prudence when granting loans that carry a higher degree of risk, thereby restricting the level of credit risk involved [59]. The relationship between liquidity and credit risk presents a particularly complex challenge for Islamic banks. Islamic banks are required to maintain a larger liquidity reserve due to the limited availability of liquidity assets in financial markets, thus providing a limited cushion. This restricts their ability to invest in ventures that are considered risky [60]. However, Islam [56] indicated that banks with lower liquidity have less credit risk, while Kharabsheh [54] found no effect for bank liquidity on credit risk. We posit that:

H_3_: Highly liquid banks–Islamic and conventional—have low credit risks. The negative effect is stronger for Islamic banks.

#### 2.2.4 Bank capital

Capital serves as the best cushion against potential financial hazards. The capital ratio estimates the proportion of a bank’s available capital to its overall assets. A high capital ratio is advantageous for banks as it enhances their capacity to absorb financial shocks, encompassing both anticipated and unanticipated significant losses arising from lending activities to borrowers [61]. Capital adequacy is a critical element of the banks’ stability and solidarity as it reflects the capital amount compared to bank loans and other assets [62].

Islam [56] indicated that banks with adequate capital effectively manage credit risk. Swami et al. [51] also pointed out that banks with low capital level have high credit risks. According to Diamond’s [63] recommendation, a higher proportion of capital leads to greater control and reduces the risk of social conformity. A decline in the quality of financing leads to a rise in provisions for losses due to defaults and an increase in implicit credit risk. This can be mitigated by capital, thereby capital negatively impacting credit risk. As per the studies conducted by Bitar et al. [64], Safiullah and Shamsuddin [9], and Trad et al. [65], it can be inferred that an increase in the capital adequacy ratio will result in a decrease in credit risk. However, the findings of Kharabsheh [54] and Muhammed et al. [2023] revealed a positive relationship between credit risk and capital adequacy.

Akhter et al. [66] argue that Islamic banks exhibit lower credit risk as a result of the disparities between asset- and credit-based financing. Errico and Sundararajan [13] and Johnes et al. [67] suggest that Islamic banks exhibit greater credit risk due to their obligation to distribute losses to depositors, especially in unfavourable economic circumstances. Therefore, Islamic banks may choose to augment their capital reserves in response to macroeconomic and institutional factors that lead to an increase in the non-performing loan (NPL) ratio. Our hypothesis posits that:

H_4_: Banks both Islamic and conventional with larger capital ratio have lower credit risk. The negative effect is stronger for Islamic banks.

#### 2.2.5. Quality of assets

Quality of assets is one of the most important indicators of the overall condition of a bank. It is affected mainly by the quality of the loan portfolio and its credit management but also by other real estate, other investments, off-balance sheet items and, to a lesser extent, cash and fixed assets [26]. Financial institutions use asset quality to manage the likelihood of loan or lease default and assess the marketability of the assets in which they invest. The quality of assets has a significant impact on various financial aspects such as profitability, liquidity, and expenses. The exposure to credit risk is influenced by the quality of assets as it affects the market pricing of the asset when it is sold to a third party. According to Swamy [68], financial institutions are obligated to promptly detect and address any inferior assets in order to implement precautionary measures.

The deterioration of asset quality, the ratio of loan loss provisions to total assets, can result in non-performing loans (NPLs), which have the potential to negatively impact a bank’s financial standing [1,9]. According to the studies conducted by Salas and Saurina [52] and Castro [69], there exists a positive correlation between the substandard quality of assets and the extent of non-performing loans. Poudel [70] asserts that variation in the level of asset quality have a positive effect on the magnitude of the projected loan loss provision. According to Abedifar et al. [1], an elevated percentage of loan loss provision would augment the likelihood of insolvency risk for the bank. Thus, the present research postulates that:

H_5_: Banks–both Islamic and conventional—with lower quality of assets have higher credit risk. The negative effect is stronger for Islamic banks.

#### 2.2.6 Type of bank: Conventional versus Islamic

The MENA region is recognized as the second largest region in the provision of Islamic banking services [17]. The distinct dissimilarities between the two categories of banks—Islamic and conventional—and their significance in the MENA region provide a rationale for incorporating this variable into the present investigation. The utilization of profit and loss sharing in Islamic banking institutions results in a distinct credit risk and profit exposure in comparison to interest-based financing in conventional banking institutions [48]. Lassoued [71] found that Malaysian Islamic banks are much more susceptible to credit risks compared to the conventional banks. The high credit risk of the Islamic banks may be attributed the usage of profit loss sharing. Findings in How et al. [49] indicate that Islamic banks exhibit lower levels of credit risk as compared to their conventional counterparts. However, the sense of competition between the two groups of banks may lead to excessive risk-taking and insolvency [4]. As discussed earlier, due to multi-layered supervision, Islamic banks are backed up by the moral ground, leaving them with a limited cushioning system available to conventional banks. Hence, Islamic banks rely on internal filtering systems to reduce credit risks rather than depending on any reactive mechanisms, such as earnings management using loan loss provisions [31]. However, smaller size and limited liquidity do not often support Islamic financial system that contribute to higher credit risk for Islamic banks.

H6: The Islamic banks will have higher credit risk exposure than their conventional counterparts.

### 2.3 Macroeconomic variables

#### 2.3.1. GDP growth rate

A higher rate of GDP growth is indicative of improved investment opportunities and increased consumer demand, which in turn contribute to a sound financial performance for credit clients. In the event of economic growth, financial institutions perceive a reduced likelihood of default, resulting in a decrease in non-performing loans (NPLs) [72]. Consistently, Karaaslan [73] and Islam [56] identified a negative correlation between the GDP and the NPL. A higher rate of GDP growth signifies that depositors are able to engage in long-term investments, thereby reducing time constraints on banks. Consequently, banks are able to provide borrowers with more time to repay loans. The implementation of this procedure is expected to result in a decrease in the overall credit risk. Thus, we expect low credit risk during high economic growth. However, Antony and Suresh [46] pointed out that GDP is positively associated with credit risk while Kharabsheh [54] and Muhammed et al. [3] found that GDP has no significant impact on credit risk.

#### 2.3.2. Regulatory quality

The banking sector is subject to stringent regulations. According to Okafor and Fadul [14], the implementation of prudential regulation plays a crucial role in ensuring stability within the financial sector by overseeing banks and preventing them from engaging in excessive risk-taking. According to Gentzoglanis [74], the implementation of regulations serves as a means for banks to operate securely and mitigate the negative effects of excessive competition within the industry. We expect that the strong regulatory quality will reduce the extent of credit risk.

#### 2.3.3. Inflation

The phenomenon of inflation is known to have a negative impact on purchasing power while simultaneously leading to an increase in the cost of credit. During a period of high inflation, borrowers may encounter challenges in meeting their loan repayment obligations, leading to an elevation of credit risk. Muhammed et al. [3] revealed a significant positive relationship between credit risk inflation. Wiryono and Effendi [75] investigated the effect of the inflation rate on non-performing loans (NPL) and revealed a significant negative correlation. The findings suggest that an increase in inflation would lead to a decrease in non-performing loans (NPL), which is unexpected from a theoretical standpoint. Antony and Suresh [46] and Islam [56] reported that inflation adversely impacts credit risk. During high inflation, banks will prioritize the most essential sectors for credits that are the most trustworthy and stable. This will reduce the amount of credit disbursement as well as credit risk. However, Havidz and Setiawan [76] and Kharabsheh [54] documented an insignificant relationship between the inflation rate and credit risk. Based on the credit constraints applied by most banks during high inflation periods, we expect a negative relationship between the inflation rate and credit risk.

#### 2.3.4. Unemployment

The unemployment rate is a significant factor in determining the credit risk of banks, since it directly affects the loans provided [77]. An increased unemployment rate suggests a decline in clients’ cashflow, which in turn affects their ability to repay debts [54]. Aydemir et al. [78] observed that the impact of the unemployment rate on credit risk varies according on the kind of bank. They found that participatory banks in Turkey were more susceptible to the influence of unemployment. Irrespective of type of banks, most empirical studies, such as Kharabsheh [54], Karaaslan [73], and Al Masud and Hossain [79], have shown a positive correlation between unemployment and non-performing loans (NPL).

## 3. Research design

### 3.1 Data and measurement of variables

The data pertaining to credit risk and bank-specific variables was obtained from Orbis BankFocus (Bureau van Dijk) database. The macroeconomic factors were collected from the World Bank database and the type of the bank is hand coded based on the website of the bank. The dataset originally downloaded contains 582 banks and other financial institutions including leasing firms, finance companies and stock exchanges for the period 2006 to 2021 from MENA region. We first filtered the data for any nonbank institution. Second, we filtered the data for missing “non-performing loans” variable as it is the main interest of this study. Finally, we checked the variables for any inconsistences, obvious outliers, or less than three consecutive observations. The final set of data utilized in this study is an unbalanced panel of 320 banks operating in 20 countries within the MENA region (Algeria, Bahrain, Egypt, Iran, Iraq, Israel, Jordan, Kuwait, Lebanon, Libya, Malta, Morocco, Oman, Qatar, Saudi Arabia, Syrian, Tunisia, United Arab Emirates, Palestine, and Yemen) during the period spanning from 2006 to 2021. The final set of firm-year observations was 2488 consisted of 1953 of conventional banks and 535 of Islamic banks. While the data was wonsorized at 1 and 99%, we did not find any significant difference in the results before and after the treatment for outliers.

Table 1 presents a list of the dependent and independent variables employed in various models accompanied with their definition and references. According to Dimitrios et al. [80], credit risk refers to the uncertainty that the banks will fail to secure a full and profitable collection of credit extended to borrowers within the designated timeframe. According to various academics [48,81–83], the assessment of credit risk makes use of a ratio between non-performing loans (NPL) and total loans or total assets. The assessment of credit risk in this study followed this literature and used the ratio of non-performing loans (NPL) to total loans (NPL1) and the ratio of non-performing loans to total assets as an additional proxy (NPL2) to consider the impact of credit risk on the total investment.

**Table 1:** Measurement of variables.

Variable	Definition	References
NPL1	*First proxy for credit risk*: Non-performing loans to gross loans and advances granted to customers	[48,54,72,81–83]
NPL2	*Alternative proxy for credit risk*: Total non-performing loans divided by the total assets	[46,84–86]
SIZE	*Size of the banks*: Natural logarithm of total asset	[46,49,57,72]
PROF	*Profitability of the banks*: Return on assets by taking the percentage of net profit after tax to total assets	[2,54,64,65,72]
LIQ	*Liquidity of the banks*: Total loans divided by total deposits.	[2,9]
CAR	*Capital adequacy ratio*: A ratio of the total equity to total asset.	[54,64,65,72,87]
ASQ	*Asset quality*: Ratio of loan loss provisions to total loans. Note: this variable increases with the poor quality of assets and should be interpreted accordingly.	[49,88]
GDP	*Economic growth*: Measured as the percentage change in real gross domestic products	[46,48,54,65,72,84,85]
INF	*Inflation rate*: Price change throughout the economy using GDP deflator	[46,48,65,84,85]
REQ	*Regulatory quality*: It measures the ability of the government to formulate and implement sound policies and regulations that help foster the private sector.	[89] and Worldwide Governance Indicator.
Unemploy	*Unemployment*: The annual unemployment rate as provided by Gender Statistics of World Bank.	[54,79]
BNK	*Type of banks*: Measured as Dummy variable, where ‘1’ is used for Islamic, while ‘0’ is used for conventional.	Added to control for differences in credit risk associated with bank type.
Covid19	A dummy variable, where ‘1’ is used for years 2020 and 2021, while ‘0’ is used for other years.	Added to control for differences in credit risk associated Covid19 crisis.

The natural logarithm of total assets is used to determine bank size. The profitability of a company has been measured using the return on assets (ROA), which is calculated by dividing the net income after tax by the total assets. Rashid et al. [90] used cash-to-asset and investment-to-asset ratios as direct indicators of liquidity. These proxies fail to consider the relationship between the portfolio of assets and the portfolio of liabilities for a bank. The present study uses the loan-to-deposit ratio as a metric to consider the assets and liabilities liquidity of the financial institution. The total equity to asset ratio serves as a proxy for the capital ratio. The asset quality was measured using the proportion of loan loss provisions to the overall amount of loans. A greater proportion of provision suggests a lower quality of loans.

The GDP growth is regarded as a proxy for economic growth. Inflation indicates the rate of price change throughout the economy using GDP deflator. Both provided by World Development Indicators. The measure of regulatory quality is derived from the Worldwide Governance Indicators, which have been published utilizing the methodology established by Kaufmann et al. [89]. Unemployment is the proportion of the workforce that is unemployed but looking for work provided by Gender Statistics of World Bank.

In order to account for the distinct significance of Islamic banks within the MENA region, a dummy variable was employed to differentiate between bank types. Specifically, a value of ’1’ was assigned to represent Islamic banks, while a value of ’0’ was assigned to denote conventional banks. Finally, to account for any structural break resulted from the Covid19 pandemic, a dummy variable assigned a value of 1 for the years 2020 and 2021 and zero otherwise is utilized.

### 3.2 Models and estimation methods

The fundamental framework comprises eleven explanatory variables, while non-performing loans (NPL) is the dependent variable. Eq (1) displays the basic model.

$${NPL}_{it}=b_{0}+b_{1}{SIZE}_{it}+b_{2}{PROF}_{it}+b_{3}{LIQ}_{it}+b_{4}{CAR}_{it}+b_{5}{ASQ}_{it}+b_{6}{GDP}_{t}+b_{7}{INF}_{t}+b_{8}{REQ}_{t}+b_{9}{Unemploy}_{t}+b_{10}{BNK}_{it}+b_{11}{Covid19}_{t}+\varepsilon_{it}$$ Eq (1)

where the notations are used for non-performing loans (NPL), bank size (SIZE), profitability (PROF), liquidity (LIQ), capital ratio (CAR), asset quality (ASQ), GDP growth (GDP), inflation (INF), regulatory quality (REQ), (Unemploy) unemployment, bank type (BNK), and (Covid19) is the Covid 19 dummy. Subscripts, such as ‘*i*’ is used for the cross-section, ‘*t*’ is for the time, and *ε_it_* is used for the error term. Our estimation methodology has been tailored to panel data techniques, taking into account the data structure. We assessed the presence of fixed and random effects through the application of the Hausman test. According to Hausman and Taylor’s [91] assertion, the null hypothesis posits that the random effect models exhibit efficiency. We found that fixed effects provide the efficient specifications. We checked for multicollinearity using VIF which indicates the absence of this problem. Finally, we used robust standard errors to account for the effect of heteroscedasticity.

In the second stage, we have introduced several interaction terms by multiplying the type of bank (BNK: Islamic versus conventional) with other bank level variables. Islamic banks are generally smaller, less diversified, and less profitable than conventional banks [90]. Islamic banks credit management passes through several layers of rigorous supervision, which differentiates their asset quality from that of conventional banks [32]. Hence, we expect that the interaction terms will help differentiate the credit risk between Islamic and conventional banks. A modification of the basic model is shown in Eq (2).


$${NPL}_{it}=b_{0}+b_{1}{SIZE}_{it}+b_{2}{PROF}_{it}+b_{3}{LIQ}_{it}+b_{4}{CAR}_{it}+b_{5}{ASQ}_{it}+b_{6}{GDP}_{t}+b_{7}{INF}_{t}+b_{8}{REQ}_{t}+b_{9}{Unemploy}_{t}+b_{10}{BNK}_{it}+b_{11}{Covid19}_{t}+b_{10}{SIZE*BNK}_{it}+b_{11}{PROF*BNK}_{it}+b_{12}{LIQ*BNK}_{it}+b_{13}{CAR*BNK}_{it}+b_{14}{ASQ*BNK}_{it}+\varepsilon_{it}.$$ Eq (2)

Finally, despite the belief that credit risk is generally static over time and that tests using ordinary least squares (OLS) are suggested to estimate the empirical relationship [92], this study has employed several dynamic models to investigate the dynamism of these models. Since lagged dependent variables are likely to be endogenous [93,94] (Arellano and Bond, 1991; Bond, 2002), a Generalized Method of Moments (GMM) approach was used to overcome this endogeneity. Estimates of GMM are generally consistent and have the large sample properties of conversion to the true value in large data samples [95]. GMM can mitigate the endogeneity problem by using internal instruments, which allow the estimates to be consistent. Arellano and Bover [96] and Blundell and Bond [97] propose the System Generalized Methods of Moments (System-GMM) as an alternative to First-differenced GMM. The System-GMM estimator is derived from the estimation of two types of simultaneous equations: one in the first difference and the other in the level. Blundell and Bond [97] use the lagged level to instrument the first difference and the lagged first difference to instrument the level. The System-GMM estimator is found to be more efficient and has a less finite sample bias due to the exploitation of more moment conditions, especially when the instruments are weak. Bond [93] suggests that two-step GMM has better properties in the context of persistent series, which is likely to be the case in this research.

## 4. Results and discussion

We discussed the results in four sections. The first section includes descriptive statistics and correlation analysis. The second section includes estimates using OLS and fixed effect models. The third section includes results on dynamic models using System-GMM. The fourth section includes summary discussions of the results.

### 4.1 Descriptive statistics

According to Table 2, the average NPL1 is approximately 10.1% (with an average NPL2 of approximately 4%), indicating that a proportion of approximately 10% of loans (or 4% of assets) are not repaid to the bank within the designated timeframe. The mean return on assets (ROA), which serves as a proxy for assessing profitability, is 1.5%. The loans-to-deposit ratio, which is a measure of total liquidity, is 0.875. The mean proportion of equity in relation to assets is 15.5%. More banks operate within the conventional domain where Islamic banks are only 21.5% of the sample. The mean annual growth in GDP is 2.6%, while the mean rate of inflation is 6.2% and unemployment is 7.7%. The regulatory quality in the MENA region was assessed on a scale ranging from -2.5 to +2.5. The mean regulatory quality was found to be 0.003, which is low even positive. However, it is noteworthy that the standard deviation of this variable is high reflecting the variations between countries in the region un terms regulatory quality. The descriptive results of our study exhibit a significant degree of similarity to those of Mousa and Zaiani [98].

**Table 2:** Descriptive statistics.

Variable	Obs	Mean	Std. Dev.	Min	Max
NPL1	2488	0.101	0.156	0.000	1.525
NPL2	2488	0.040	0.052	0.000	0.507
SIZE	2488	21.241	2.931	10.941	26.191
PROF	2488	0.015	0.028	-0.279	0.482
ASQ	2443	0.077	0.110	0.000	1.000
LIQ	2468	0.875	6.170	0.000	193.834
CAR	2488	0.155	0.121	-0.070	0.966
BNK	2488	0.215	0.411	0.000	1.000
GDP	2478	0.026	0.052	-0.280	0.262
INF	2478	0.062	0.127	-0.302	1.500
REQ	2488	0.003	0.768	-2.249	1.334
Unemploy	2488	0.077	0.057	0.001	0.264
Covid19	2488	0.107	0.309	0.000	1.000

Notes: NPL1 = Share of Non-performing loans to total loans, NPL2 = Share of Non-performing loans to total assets, SIZE = bank size in LN of total assets, PROF = return on asset, ASQ = Share of loan loss provision to total loans, LIQ = total loans over total deposit, CAR = share of bank equity to total assets, BNK = dummy variable (1 for Islamic banks), GDP = GDP growth rate, INF = inflation rate, REQ = Regulatory quality, and Unemploy = Unemployment rate published by the World Bank. Covid19 = dummy for years 2020 and 2021.

The correlation coefficient matrix is presented in Table 3. The table did not reveal any high correlations between independent variables, with the exception of a negative 0.435 coefficient observed between the inflation rate and regulatory quality and a negative 0.432 between asset quality and regulatory quality. One or both of NPL proxies exhibited an adverse correlation with bank size, GDP growth rate, and regulatory quality, while a positive correlation was observed with asset quality, inflation, unemployment and capital adequacy. The relationship between the profitability and liquidity with each proxy for non-performing loans exhibits varying outcomes. Theoretically, the majority of these relationships are anticipated. The correlation coefficients obtained in our study were consistent with those reported in other relevant studies pertaining to the banking industry [48,99].

**Table 3:** Correlation matrix.

Variables	NPL1	NPL2	SIZE	PROF	ASQ	LIQ	CAR	GDP	INF	REQ	Unemploy
NPL1	1.000										
NPL2	0.684	1.000									
SIZE	-0.269	-0.215	1.000								
PROF	0.097	-0.025	-0.116	1.000							
ASQ	0.824	0.517	-0.267	0.131	1.000						
LIQ	-0.004	0.094	-0.106	-0.012	-0.011	1.000					
CAR	0.265	0.059	-0.272	0.270	0.257	0.245	1.000				
GDP	-0.330	-0.235	0.180	-0.044	-0.322	-0.018	-0.018	1.000			
INF	0.272	0.176	-0.206	0.185	0.273	-0.050	-0.036	-0.292	1.000		
REQ	-0.436	-0.270	0.286	-0.198	-0.432	0.037	-0.079	0.403	-0.435	1.000	
Unemploy	0.180	0.130	-0.286	0.003	0.155	-0.033	-0.092	-0.168	0.145	-0.511	1.000

### 4.2 Static determinants of credit risks

We have tested for the right model between fixed and random effects using the Hausman Test. The null hypothesis of the test, that ‘the random effect is efficient, was rejected. Therefore, we proceeded with the fixed-effect models. Panel data methods with the fixed effects estimator can be used to control for variables that are common to all firms but not included in the regression [100]. Models 1, 2, and 3 use NPL2, and Models 4, 5, and 6 use NPL1 to proxy credit risk. The pooled OLS for each proxy are first estimated for comparison.

The outcomes of pooled ordinary least squares (Models 1 and 4) and fixed effects models (2 and 5), which incorporate firm and country fixed effects, are presented in Table 4. The relationships in the OLS and fixed effect outcomes exhibit similarity for most variables. The size of the bank, its profitability, and GDP growth all have a negative impact on credit risk. Stated differently, banks that are characterized by larger size and profitability exhibit lower levels of credit risk and those work in higher GDP growth countries also face lower credit risk. Conversely, banks that maintain a higher level of loan provisions (poor asset quality) are perceived as having higher credit risk. The results are mostly positive for liquidity but the effect is statistically insignificant in most cases. The effect of capital adequacy is mostly positive as well, meaning that higher capital reserve will help reduce credit risk.

**Table 4:** Determinants of credit risk.

	(1)	(2)	(3)	(4)	(5)	(6)
VARIABLES	NPL2	NPL2	NPL2	NPL1	NPL1	NPL1
	OLS	FE	FE(int.)	OLS	FE	FE(int.)
SIZE	-0.00413**	-0.000922	-0.00104	-0.00301*	-0.000871	-0.00133
	(0.00162)	(0.000581)	(0.000604)	(0.00150)	(0.000930)	(0.00101)
PROF	-0.207*	-0.254***	-0.248**	-0.379*	-0.312**	-0.266**
	(0.104)	(0.0791)	(0.0858)	(0.200)	(0.125)	(0.125)
ASQ	0.225***	0.232***	0.242***	1.046***	0.945***	0.939***
	(0.0235)	(0.0399)	(0.0354)	(0.0604)	(0.0755)	(0.0667)
LIQ	0.000883***	0.000237	0.000242	-0.000359*	0.000137	0.000146
	(0.000202)	(0.000200)	(0.000191)	(0.000172)	(0.000476)	(0.000449)
CAR	-0.0515***	0.0360*	0.0385*	0.0938**	0.121**	0.128***
	(0.0130)	(0.0183)	(0.0196)	(0.0352)	(0.0508)	(0.0418)
BNK	-0.00602**	-0.0265**	0.0271	-0.00826***	-0.236**	-0.270**
	(0.00207)	(0.00992)	(0.0161)	(0.00270)	(0.0888)	(0.0991)
SIZE*BNK			0.000490			0.00185*
			(0.000398)			(0.000900)
PROF*BNK			-0.0393			-0.268*
			(0.168)			(0.145)
LIQ*BNK			-0.0245***			-0.0677***
			(0.00817)			(0.0118)
CAR*BNK			-0.00264			0.00150
			(0.0340)			(0.0836)
ASQ*BNK			-0.0533*			0.00948
			(0.0661)			(0.120)
GDP	-0.0571*	-0.0762***	-0.0761***	-0.148***	-0.110**	-0.109**
	(0.0287)	(0.0138)	(0.0136)	(0.0454)	(0.0406)	(0.0397)
INF	0.0181	-0.00170	-0.00147	0.0550**	0.0253	0.0249
	(0.0137)	(0.0109)	(0.0108)	(0.0198)	(0.0211)	(0.0204)
REQ	-0.00119	0.00589	0.00709	-0.0143***	-0.00796	-0.00604
	(0.000946)	(0.00636)	(0.00638)	(0.00394)	(0.0100)	(0.00900)
Unemploy	-0.0311	0.0142	0.0175	0.0300	-0.0325	-0.0239
	(0.0196)	(0.0408)	(0.0409)	(0.0281)	(0.0947)	(0.0952)
Covid19	-0.0301**	-0.0109**	-0.0113**	-0.0304**	-0.0185**	-0.0192***
	(0.0109)	(0.00436)	(0.00433)	(0.0105)	(0.00639)	(0.00593)
C	0.129***	0.0386**	0.0398**	0.0786**	0.0281	0.0360
	(0.0391)	(0.0141)	(0.0149)	(0.0362)	(0.0247)	(0.0272)
Observations	2,414	2,414	2,414	2,414	2,414	2,414
R-squared	0.321	0.795	0.796	0.698	0.880	0.881
Bank dummy	No	Yes	Yes	No	Yes	Yes
Country dummy	No	Yes	Yes	No	Yes	Yes

Notes: Dependent variable is NPL. Notes: NPL = Share of Non-performing loans to total assets, SIZE = bank size in LN of total assets, PROF = return on asset, ASQ = Share of loan loss provision to total loans, LIQ = total loans over the total deposit, CAR = share of bank equity to total assets, BNK = dummy variable (1 for Islamic banks), GDP = GDP growth rate, INF = inflation rate, and REQ = Regulatory quality published by the World Bank. Covid19 is a dummy for years affected by Covid-19 pandemic. Standard errors in parenthesis. Significance levels are denoted as *** = p<0.01, ** = p<0.05, * = p<0.1. Results are robust by White cross-section standard errors and covariance (*d*. *f*. corrected). FE (int.) = Fixed effect with interaction terms.

The results identify the type of bank to be a significant indicator. Consistent with our conjecture, Islamic banks have had lower NPLs than conventional banks. Apparently, due to the smaller amount of loans relative to conventional banks, Islamic banks failed to enjoy the benefits of economies of scale. It is worth mentioning here that Islamic banks globally are smaller in size compared to conventional banks. Some support for the effect of improvement in regulatory quality on reducing the NPL is found. The quality of regulations serves as a means for banks to operate securely and mitigate the negative effects of excessive competition within the industry [74]. The effect of inflation is mostly insignificant except for Model 4 where it is positive. During periods of high inflation, banks tend to adopt a prudent approach towards credit disbursement, which can result in a reduction in NPLs [75]. The unemployment is insignificant for all models. Surprisingly, the Covid19 variable has a negative effect on NPL for all models.

The results of including interaction terms in Models 3 and 6 is also highlighted in Table 4. The interaction of bank type with liquidity and asset quality has a negative impact on non-performing loans (NPL). The interaction terms’ negative coefficients suggest that the effects of these two variables are lower for Islamic banks’ than they do on conventional banks. The aggregate effect is the sum of the variable’s coefficient and the interaction term’s coefficient.

### 4.3 Dynamic analysis using 2-step System-GMM

Table 5 displays the outcomes obtained from the utilization of the 2-step System-GMM. Models 1 and 3 estimate the main determinants, while Models 2 and 4 add the interaction terms. Many studies have indicated that the factors influencing credit risk are typically robust in a static model [92], thereby partially refuting the importance of a dynamic analysis. The results of the System-GMM models yield two significant conclusions. First, the lagged NPL exhibits a significant effect on the current NPL, indicating persistence of the NPL, particularly in the case of NPL2 where the NPL is standardized by total assets. Das and Ghosh [83] have reported comparable findings on Indian banks, while Castro [69] has documented analogous outcomes on banks originating from Greece, Ireland, Portugal, Spain, and Italy. In the present scenario, the lagged credit risk variable assumes greater significance than all other variables in the process of short-term adjustment. Profitability, inflation, unemployment, and GDP growth exhibited significant negative effect with NPL2. GDP growth is also significant with NPL1 with the expected negative sign similar to Barra and Ruggiero [72]. The asset quality has positively affected NPL1. The bank type negatively affects NPL1 indicating lower level of credit risk of Islamic banks. Other variables are mostly insignificant.

**Table 5:** Determinants of credit risk analysis using 2-step system GMM.

	(1)	(2)	(3)	(4)
VARIABLES	NPL2	NPL2	NPL1	NPL1
	Sys-GMM	Sys-GMM(int.)	Sys-GMM	Sys-GMM(int.)
NPL1 (-1)	0.939***	0.941***		
	(0.105)	(0.103)		
NPL2 (-1)			0.429**	0.484***
			(0.184)	(0.176)
SIZE	-0.000	-0.000	-0.000	-0.001
	(0.001)	(0.001)	(0.002)	(0.002)
PROF	-0.077*	-0.062	-0.075	0.030
	(0.045)	(0.046)	(0.173)	(0.180)
ASQ	0.017	0.016	0.756***	0.621***
	(0.020)	(0.022)	(0.200)	(0.215)
LIQ	0.0001**	0.0001**	-0.0001	-0.0001
	(0.000)	(0.000)	(0.000)	(0.000)
CAR	0.001	0.001	0.036	0.031
	(0.008)	(0.009)	(0.030)	(0.030)
BNK	-0.002	0.010	-0.003	-0.085**
	(0.002)	(0.013)	(0.004)	(0.038)
SIZE*BNK		-0.001		0.003*
		(0.001)		(0.002)
PROF*BNK		-0.136		-0.388
		(0.116)		(0.356)
LIQ*BNK		0.009		0.028
		(0.008)		(0.041)
CAR*BNK		-0.008		-0.044
		(0.015)		(0.082)
ASQ*BNK		0.008		0.262
		(0.019)		(0.183)
GDP	-0.097***	-0.096***	-0.169***	-0.184***
	(0.024)	(0.024)	(0.065)	(0.067)
INF	-0.014**	-0.015***	0.006	0.003
	(0.006)	(0.006)	(0.012)	(0.012)
REQ	0.000	0.000	0.001	0.000
	(0.001)	(0.001)	(0.004)	(0.004)
Unemploy	-0.024**	-0.023**	0.004	0.009
	(0.010)	(0.010)	(0.032)	(0.036)
Covid19	-0.010*	-0.009*	-0.013	-0.013
	(0.005)	(0.005)	(0.014)	(0.015)
Constant	0.015	0.011	0.006	0.024
	(0.019)	(0.020)	(0.043)	(0.044)
Obs.	1,961	1,961	1,961	1,961
Banks No.	287	287	287	287
AR(1) (*p*)	0.00475	0.00495	0.100	0.0848
AR(2) (*p*)	0.404	0.431	0.942	0.968
Hansen (*p*)	0.199	0.213	0.256	0.228

Notes: Standard errors are in parentheses. Significance levels are denoted as *** = p<0.01, ** = p<0.05, * = p<0.1. AR (1) and AR (2) are the significance levels for Arellano-Bond serial correlation tests. Hansen test statistics significance level indicate the over-identifying restrictions.

### 4.4 Discussions of the results

In line with a burgeoning body of research [11,83,101], our findings indicate that large banks exhibit minimal credit risk. This holds true for both Islamic and conventional banks. Small Islamic banks have superior financial stability in comparison to small conventional banks [88]. Likewise, banks that are highly profitable are less likely to provide risky loans to borrowers. Our contention is that, although being extremely lucrative and possessing valuable assets, big banks in the MENA area do not partake in hazardous loans due to the predominant Islamic moral practises. Islamic banks, in particular, strive to avoid making risky loan, while having a larger cushion in Islamic nations compared to regular banks. Thus, the primary and the biggest distinguishing element in this context is the adherence to Islamic religious customs.

There have been several instances when increased liquidity has been associated with elevated levels of risk-taking [102]. During periods of heightened credit risk, banks make efforts to reduce their exposure to risky credit, even when there is an abundance of loanable funds [103]. Islamic banks uphold a large size of liquidity as a result of the restricted availability of Shariah-compliant financing choices in the market. According to Olson and Zoubi [59], banks in the GCC area maintain a greater level of liquidity as a result of their managerial actions, rather than due to a scarcity of investment options. This also proves that Islamic managerial practices prohibit making benefits by taking extra credit risk using surplus liquidity.

We have found a strong positive correlation between the substandard quality of assets and credit risk, which is supported by several past studies [52]. The long-term financial prospects of a bank will be adversely impacted by a decline in the quality of its assets [70]. Islamic banks, even if they have a better financial position in a collective culture, are very sincere in managing the quality of their assets. Islamic banks assess the ability of borrowers to handle risk in the event of unexpected changes in cash flows, rather than just relying on projected future cash flows. This is because the financing process involves active participation from both lenders and borrowers [31,90]. The application of this conservative management strategy, in conjunction with adherence to Islamic moral principles, effectively mitigates the credit risk. Conventional banks often prioritise a larger credit risk in order to get a greater return, but Islamic banks employ stringent control systems to avoid excessive risk; earning profit is not the primary objective of an Islamic bank [15].

Consistent with previous research, there is a negative correlation between inflation rate and credit risk in the dynamic models [2,75,99]. As a result of the elevated inflation rate, especially during extended periods caused by significant regional crises such as the Arab Spring, banks in the MENA area implement stringent regulations to mitigate risky investments owing to economic concerns. Similar actions were undertaken during the global financial crisis and the Covid-19 pandemic. As a result, Islamic banks were found to be resilient to global financial crisis.

A negative correlation between the quality of regulations and the level of credit risk highlights the need of having a high-quality legal framework. Regulatory quality refers to the government’s capacity to develop and enforce efficient rules and regulations that support the long-term expansion of the private sector. The MENA area had subpar regulatory quality, despite the efforts of several nations to implement smart policies. Inadequate regulatory standards in a prominent Islamic nation will have an adverse impact on the risk management framework in Islamic banks. Without stringent regulatory oversight, Islamic banks may attempt to circumvent the Shariah screening processes, potentially resulting in increased risk.

Nawary et al. [104] argued that banks must implement robust credit management strategies in order to prevent insolvency. Profitability, liquidity, and asset quality have a greater impact on reducing credit risk for Islamic banks compared to conventional banks. Islamic banking practises commonly employ asset-based financing, requiring Islamic banks to carefully assess each funding request before granting approval to clients. Thus, it is expected that Islamic banks will demonstrate exceptional asset quality, thereby mitigating their credit risk. An important consequence of these findings is that Islamic banks, although having robust societal and state support, are able to mitigate credit risk due to adherence to Islamic moral requirements, stringent rules, and conservative management practises.

Abdullah et al. [105] argued that conventional banks should prioritise assets of good quality, instead of relying solely on the utility maximisation approach like traditional credit managers. Previous research has indicated that there are no significant variations in the factors that contribute to credit risk between Islamic and conventional banks [106]. However, our findings demonstrate that Islamic banks, despite having a more robust financial cushion, are able to mitigate credit risk due to their adherence to strict Islamic principles, which results in higher asset quality.

## 5. Conclusion

Multiple financial crises have imparted the knowledge that banks are rescued by the use of funds provided by taxpayers. They also enjoy the depositor’s insurance program if there is a high liquidity crisis. The cushion hypothesis posits that the presence of enough safety nets leads to greater risk-taking, since the banks are assured of assistance in the event of failure. This phenomenon is considerably more pronounced in collective societies. This study concludes that Islamic banks exhibit a lower level of credit risk compared to conventional banks. Even though smaller Islamic banks are unable to take advantage of the economies of scale that comes with larger scale operations, strict ethical and moral principle in practice restrict risk-taking by the Islamic banks. The Islamic and conventional banks’ credit risk management systems are also distinguished by variables such as strong asset quality, profitability, and liquidity.

We conducted an analysis of a list of 320 Islamic and conventional banks in twenty nations within the MENA area, spanning from 2006 to 2021. In summary, our research suggests that banks that are both profitable and liquid, located in nations with a strong GDP growth, high inflation, and effective regulatory systems, tend to have a reduced likelihood of encountering credit-related issues. Profitability, liquidity, size, and asset quality have varying degrees of effect on credit risk management in Islamic and conventional banks. Islamic banks exhibit superior asset quality due to their rigorous and comprehensive monitoring system. Better asset quality has led to lower credit risk.

### 5.1 Implications

Credit risk is a significant factor contributing to both banking crises and subsequent economic crises. Minimising credit risk will have a favourable impact on the bank’s performance [107]. We discuss three relevant implications.

Firstly, the findings directly imply that depositors will feel secure when they place their funds in Islamic banks. Contrary to the cushion hypothesis, Islamic banks take lesser risk because of better monitoring and moral system, even if they could engage in high risk-taking. Enhanced asset quality and rigorous credit standards enhance the reputation of Islamic banks and lower financing costs. Despite being in collective societies with higher social cushion, better asset quality will encourage Islamic banks to engage in socially responsible lending to financially vulnerable groups, such as small and medium companies.

Secondly, conventional banks may gain valuable insights in asset quality in, and multi-layer screening system employed by Islamic banks. Islamic banks’ credit monitoring system has a higher implicit cost but can help enhance asset quality significantly. Therefore, the post-credit monitoring cost is generally very low for Islamic banks, which can be an important strategic change for the conventional banks. Islamic banks can employ artificial intelligence to reduce the pre-credit monitoring process to reduce the credit exposure further. FinTech can provide an excellent vehicle to reduce monitoring cost, increase asset quality, and reduce non-performing loans [108,109].

Thirdly, due to the lack of a worldwide liquidity market, Islamic banks maintain a substantial amount of cash reserves. High cash reserves destabilise profitability. Governments in several Islamic countries offer credit guarantee schemes for risky Islamic loans (See discussion on Kafalah scheme at www.kafalah.gov.sa/en/). These initiatives and large pool of reserves may create a false sense of cushion that must be monitored. The efforts to address the liquidity issues also fall well short of the anticipated level for an Islamic market of approximately $2.10 trillion.

### 5.2 Limitations and future research

It is our contention that conservative Islamic tenets are in opposition to both societal and governmental safeguards, even if such safeguards are accessible to Islamic financial institutions. Consequently, the imposition of ethical limitations on the cushioning mechanism would prompt Islamic financial institutions to adopt more cautious strategies, emphasizing operational efficiency and implementing a robust risk monitoring system to mitigate potential risks. This would lead to better asset quality as well. In this regard, further investigation might be conducted to construct an early warning system that is based on the probable asymmetric response of several predictors of credit risk, including the ethical norms and state-level incentives for Islamic banks. Similar studies could also extend the range and scope of data into other similar relevant contexts.
